# Differences in fatigability of muscles involved in fecal continence: Potential clinical ramifications

**DOI:** 10.14814/phy2.15144

**Published:** 2021-12-19

**Authors:** Krupa Patel, Ling Mei, Elliot Yu, Mark Kern, Navjit Lehal, Francis Edeani, Patrick Sanvanson, Emily R. W. Davidson, Reza Shaker

**Affiliations:** ^1^ Division of Gastroenterology and Hepatology Medical College of Wisconsin Milwaukee Wisconsin USA; ^2^ Department of Obstetrics and Gynecology Division of Urogynecology Medical College of Wisconsin Milwaukee Wisconsin USA

**Keywords:** anorectal manometry, external anal sphincter, fecal incontinence, puborectalis muscle, vaginal manometry

## Abstract

**Introduction:**

Fatigue of the anal sphincter complex has been demonstrated using high‐resolution anorectal manometry (HRAM). However, the fatigability of individual muscles such as the external anal sphincter (EAS) and puborectalis muscles (PRM) has not been described. Vaginal manometry has been used to study contractile activity of the PRM. By applying both modalities, we attempted to differentiate the fatigability between the PRM and the EAS under different exercise conditions.

**Methods:**

We studied two groups: group 1, 12 healthy women (21 ± 2.7 years) with HRAM and group 2, 10 healthy (20 ± 3 years) women with vaginal manometry. All subjects performed 40 repetitive contractions with and without an intra‐anal resistive load. In group 1, areas under the curve (AUC) of the anal canal high‐pressure zone (HPZ) including the caudal and rostral halves were compared. In group 2, the maximum and mean pressures of the vaginal HPZ were compared.

**Results:**

The AUC decreased significantly only after repetitive contractions against a resistive load (462 ± 129 vs. 390 ± 131 mmHg‐cm, *p* = 0.02), indicating fatigue. The caudal half (EAS) decreased significantly after contractions against a load (288 ± 75 vs. 239 ± 82 mmHg‐cm, *p* = 0.02), while the rostral half (PRM) did not. The vaginal pressures (PRM) also decreased only after repetitive contractions against a load (maximum pressures, 358 ± 171 vs. 239 ± 109 mmHg, *p* = 0.02).

**Conclusions:**

The EAS and PRM both exhibit fatigue with contractions only against a resistive load. These findings may guide the development of appropriate exercise regimens to target specific muscles involved in fecal continence.

## Graphical abstract

Fecal incontinence is a prevalent and distressing disorder with a mainstay of treatment being pelvic floor muscle exercises. Fatigue is a principle of strengthening exercises. Individual continence muscles have not been studied until this study showing the fatigability of both the external anal sphincter and puborectalis muscles.

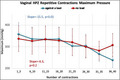

## 1 INTRODUCTION

Fecal incontinence (FI) is a debilitating and devastating disorder estimated to occur in 8% of non‐institutionalized adults and 50% of adults in nursing homes. Although these numbers are likely an under‐estimate, given the negative stigma and embarrassment associated with FI (Bharucha et al., 2015; Nelson, 2004; Whitehead et al., 2009). Conservative management is often the initial approach to treatment including patient education, fiber, anti‐diarrheal medications, behavioral techniques, and pelvic floor muscle (PFM) exercises. These efforts have shown a variable response with some improvement in symptoms and quality of life (Mazor et al., 2021; Norton et al., 2003; Rao, 2014; Whitehead et al., 2015, 2016). PFM exercises are targeted toward increasing the strength of the muscles involved in anal continence.

The anal continence complex is a group of overlapping muscles: the internal anal sphincter (IAS), the external anal sphincter (EAS), and the puborectalis muscle (PRM). The involuntary IAS maintains 70%–80% of the resting tone with the remainder contributed by the EAS (Duthie & Watts, 1965). In contrast, the EAS can contract reflexively and voluntarily. The PRM blends superiorly with the EAS. The PRM is a U‐shaped, sling‐like component of the levator ani complex that maintains the anorectal angle at rest and assists with the anal canal closure mechanism by contracting (Jung et al., 2008; Raizada et al., 2010). The EAS is located in the caudal part of the anal canal, while the PRM is more rostral. Manometric geometry of the anal canal high‐pressure zone (HPZ) reflects the distribution and contractile function of these muscles. Recent studies using high‐definition anorectal manometry (HDAM) have described axial and circumferential asymmetry of the anal sphincter HPZ, which is related to the differential contributions of the PRM and EAS (Raizada et al., 2011). Though HDAM with closely spaced circumferential sensors extending across the anal canal may permit assessment of individual muscles, it is not widely available and its accuracy in evaluating specific muscle function is still preliminary (Lee et al., 2013). Distinction of the pressures developed by the PRM and EAS muscles by high‐resolution anorectal manometry (HRAM) is difficult. However, studies have shown that PRM contraction is recordable from the vaginal canal (Guaderrama et al., 2005; Jung et al., 2007). This finding has been demonstrated with the use of 3D ultrasound and magnetic resonance imaging (MRI) in conjunction with high‐definition manometry in the vaginal canal assessing the vaginal high‐pressure zone (Raizada et al., 2010).

Most PFM exercises, such as Kegel exercises, emphasize strength training without introducing resistance. However, resistance leads to overloading the muscles, inducing neuromuscular fatigue which is a paramount principle of rehabilitative strength training (Agrawal et al., 2018; Marques et al., 2010; McArdle & Katch, 2010; Shaker et al., 2002, 2016). Our lab has created a novel device, the continence muscles Resistance Exerciser Device (cRED), that provides a resistive load during PFM exercises to allow for overloading of the continence muscles with a contraction. Previous work with this device has shown that repetitive short squeeze contractions against a resistive load successfully induced fatigue as opposed to similar contractions without a load (Mei et al., 2021). However, the fatigability of individual muscles within the anal complex has not yet been investigated.

In this work, we attempted to differentiate the fatigability between the PRM and EAS with and without resistive loads using high‐resolution anorectal and vaginal manometry under different exercise conditions. We evaluated the impact of exercise timing (beginning and end of a series of contractions) as well as the impact of adding the cRED resistive load.

## 2 MATERIALS AND METHODS

This was a prospective study using two groups of healthy women. The volunteers were all recruited by word of mouth or flyers posted in college/graduate school campuses. All volunteers denied any history of anorectal symptoms and had normal bowel habits. None of them had medical conditions or took medications that could potentially affect muscle function. One group of women was initially recruited and studied using high‐resolution anorectal manometry. Following this the other group was recruited and studied using vaginal manometry. Four participants participated in both studies. Studies were approved by the Internal Review Board of the Human Research Protection Program at the Medical College of Wisconsin (Milwaukee, WI). All participants gave written informed consent before the studies.

### 2.1 Group 1: high‐resolution anorectal manometry: 12 healthy (aged 21 ± 2.7 years) nulliparous female volunteers, with and without resistive load

#### 2.1.1 Experimental tools

##### High‐resolution anorectal manometry (HRAM)

Anorectal manometry pressures were monitored using a high‐resolution esophageal manometric catheter (ManoScan and ManoView Systems) positioned in the anal canal with the distal end in the rectum. We used the distal 8–10 sensor rows along the probe in this study.

##### Continence muscles resistance exerciser device (cRED)

**FIGURE 1:**
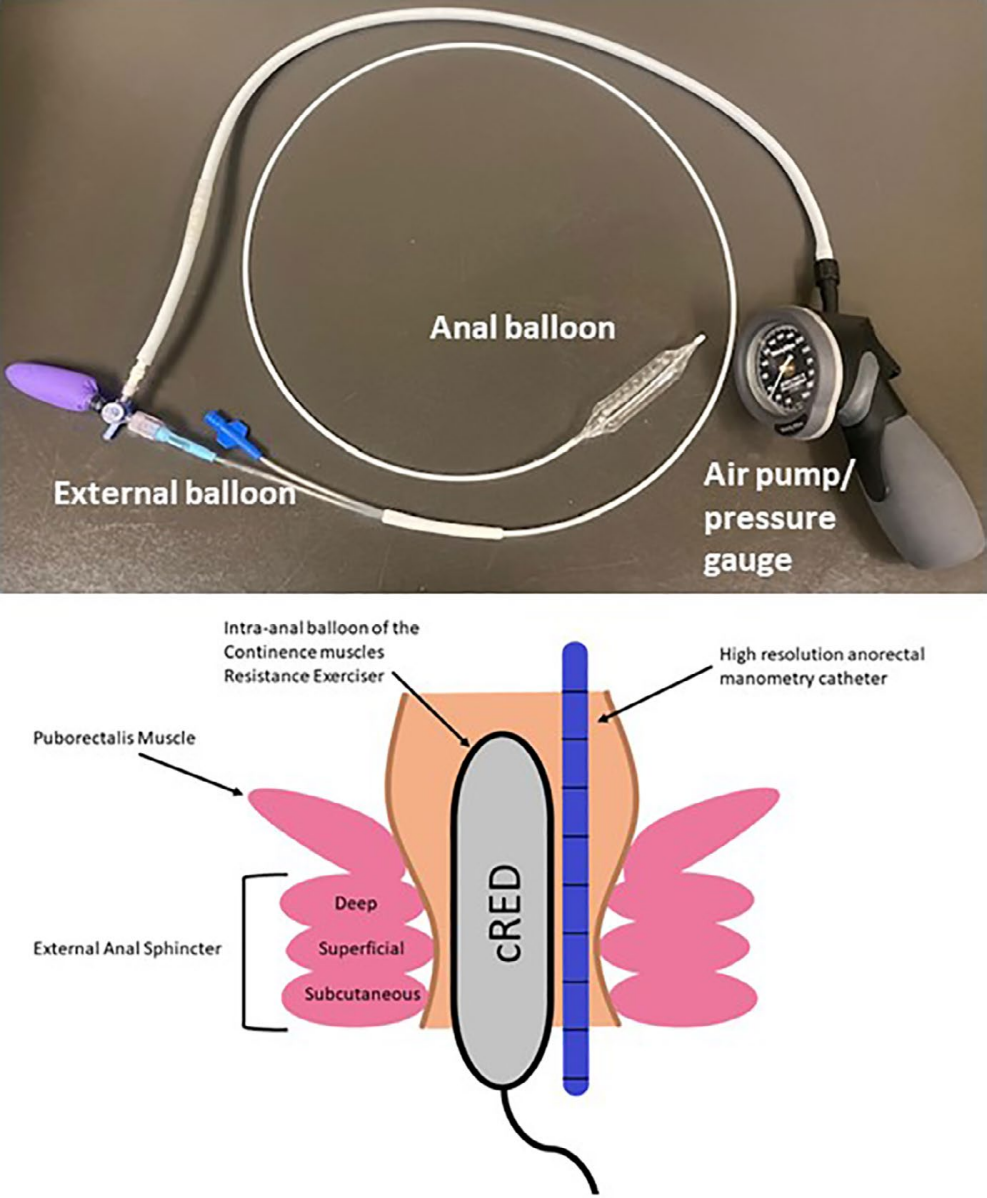
The continence muscles Resistance Exerciser Device along with a cartoon description of the device with the manometry catheter in the anal canal

Using this device, we could load the continence muscles during anal contractions (Figure 1). The cRED consists of a 6 cm × 2 cm cylindrical balloon made of non‐compliant material (intra‐anal balloon) that is connected to a small compliant balloon (external balloon) by a 2‐mm diameter tube and a stopcock to allow for air to be filled to a desired resistance of 50–60 mmHg and sealed in the system. The intra‐anal balloon, while providing resistance, is compressed by the anal contraction (exceeding 50 mmHg pressure) due to displacement of its air to the external distensible balloon. Air displacement into the external balloon results in its expansion and serves as an externally visible indicator that an anal contraction has occurred. A detailed description of this device has been previously published (Mei et al., 2021).

#### 2.1.2 Experimental protocol

All subjects were placed in the left lateral decubitus position for the duration of the anorectal manometry study. Prior to the exercises, all subjects were educated on proper pelvic squeeze technique. All subjects underwent a digital rectal examination at baseline to ensure proper squeeze technique. After applying lubricating gel along the catheter, the manometry catheter was placed inside the anal canal with the distal end in the rectum. This allowed for pressure sensors to be arranged along the length of the entire anal canal. After placement of the catheter, a 5‐min accommodation period elapsed.

Subjects were verbally cued to perform a set of exercises with and without a resistive load provided by the cRED. The order of exercises (with and without cRED) was randomized. The intra‐anal balloon of the cRED was placed in a deflated state parallel to the manometric catheter with the distal end of the balloon at or just outside the anal margin. During the exercises with the cRED, the balloon was then inflated to the desired resistance of 50–60 mmHg. Each set of exercises consisted of 40 consecutive 3‐s anal contractions alternating with 3‐s rest. The first 5 and the last 5 contractions of the 40 repetitive contractions were used for analysis.

#### 2.1.3 Manometric analysis: sample contraction is shown in Figure 2

**FIGURE 2:**
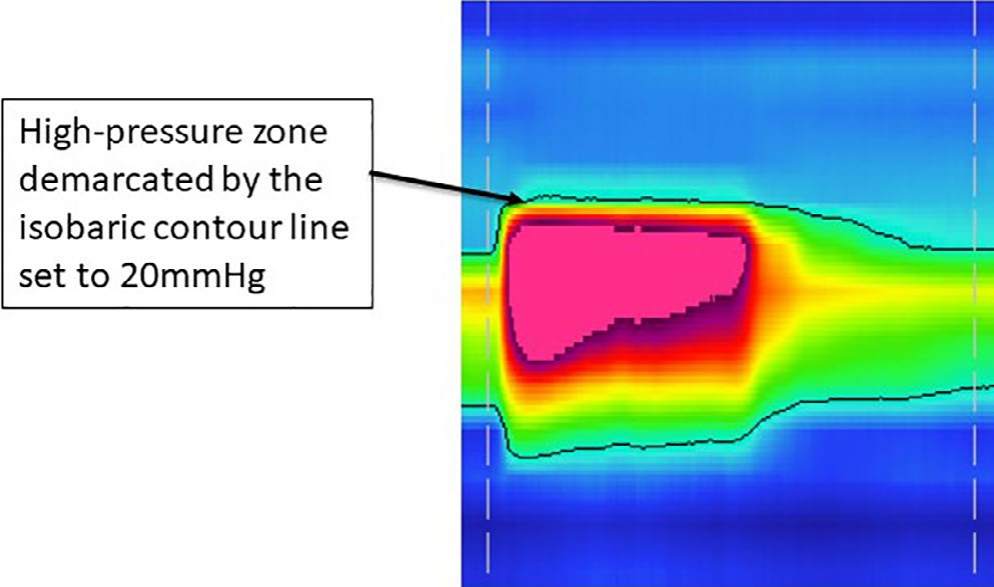
ManoView software showing a contraction on anorectal manometry by topographical color plot

##### Maximum squeeze pressures along the anal canal HPZ to determine area under the curve

Pressures were viewed on a topographical color plot with superimposed pressure tracer line graphs. Manometric parameters were obtained using the “Smart Mouse” feature on the ManoView analysis software. Maximum squeeze pressures during the 3‐s contractions were obtained for each pressure sensor within the anal canal HPZ. For the AUC calculation, we did not use interpolated data. Since there are no measured data between sensors, we used sensor data (the maximum pressure during a 3‐s squeeze) to approximate the pressure adjacent to that sensor.

#### 2.1.4 Statistical analysis

**FIGURE 3:**
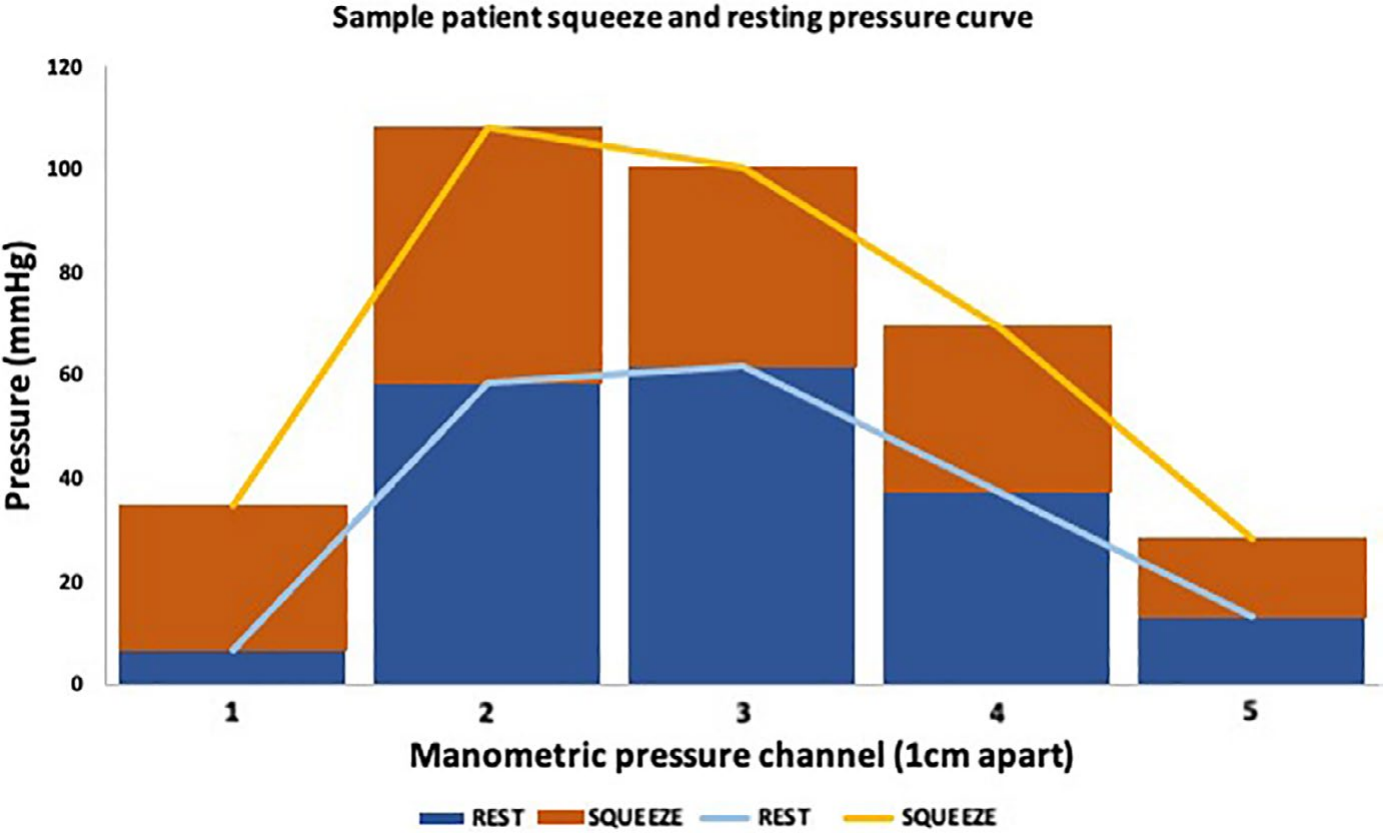
A sample patient squeeze and resting curve at a single time point during anorectal manometry. The *x*‐axis depicts the channel along the anal canal, where 1 is the channel most caudal within the anal canal. The *y*‐axis is the pressure values measured at each channel

The maximum pressure during the first five and last five contractions was averaged at each pressure sensor level within the anal canal HPZ, respectively. We created the curve by plotting pressure sensors along the *x*‐axis, 1 cm aside between adjacent sensors, and the pressure values (mmHg) along the *y*‐axis (Figure 3).

The area under the curve (AUC) was determined using the trapezoid rule. The equation for this is:

$$\frac{y_{1}+y_{2}}{2}+\frac{y_{2}+y_{3}}{2}+\frac{y_{3}+y_{4}}{2}$$

where *y_n_* is the pressure value along pressure sensor *n*.

To calculate the caudal half of the AUC, we used either of the following formulas depending on if the number of pressure sensors (*n*) within the anal canal HPZ was even or odd. If *n* was even, the equation is:

$$\frac{y_{1}+y_{2}}{2}+\frac{y_{2}+y_{3}}{2}+\left[\frac{y_{3}+\frac{y_{3}+y_{4}}{2}}{2}\right]\left[\frac{1}{2}\right]$$

where *y_n_* is the pressure along sensor *n*. If *n* was odd, we use the following equation:

$$\frac{y_{1}}{2}+y_{2}+\frac{y_{3}}{2}$$

The rostral half of the AUC was obtained by subtracting the caudal half from the total AUC.

A paired *t*‐test was used to compare the total AUC under different exercise conditions as well as the difference between the caudal and rostral halves of the AUC.

### 2.2 Group 2: vaginal manometry: 10 healthy nulliparous (20 ± 3 years) females, with and without resistive load

#### 2.2.1 Experimental tools

##### High‐resolution vaginal manometry

Vaginal manometry pressures were measured using a high‐resolution anorectal manometric catheter. The computerized recording and analysis system (Medical Measurements Systems, Laborie) store pressure data from eight sensor rows (23 pressure sensors total) spaced 1 cm aside along the probe. The manometry probe was attached to a 2.5 cm diameter hand‐made non‐compliant balloon via a sleeve anteriorly to ensure it was aligned with the vaginal wall when inserted.

#### 2.2.2 Experimental protocol

All subjects were placed in the lithotomy position for the duration of the vaginal manometry study. Prior to the exercises, all subjects were educated on proper pelvic squeeze technique. All subjects had a bimanual exam to ensure normal vaginal anatomy. The vaginal balloon with the manometry catheter in the sleeve was placed in a condom prior to placing inside the vaginal canal for proper lubrication. A 5‐min accommodation period elapsed after placement of the catheter.

Subjects then were verbally cued to perform a set of exercises with and without an anal resistive load provided by the cRED in a randomized order identical to the protocol for group 1. For analysis of vaginal manometry, the 40 repetitive contractions were grouped into 8 epochs, each consisting of 5 contractions.

#### 2.2.3 Manometric analysis

The vaginal HPZ was demarcated by setting the isobaric contour to 20 mmHg based on prior studies involving the distal esophagus (Lin et al., 2012; Mei et al., 2021), framed for each 3‐s contraction. The mean and maximum pressures of the vaginal HPZ were measured for each 3‐s frame. These pressures were then averaged for each epoch of the five contraction epochs.

#### 2.2.4 Statistical analysis

Repeated two‐way analysis of variance was used to detect decreasing mean and maximum squeeze pressure between the eight epochs. Slope values of epochs from linear regression analysis were compared for these parameters with and without the application of resistive load by the cRED using *t*‐test. Data are presented as means ± standard deviation (SD) unless stated otherwise.

## 3 RESULTS

### 3.1 Group 1—high‐resolution anorectal manometry, with and without resistive load

#### 3.1.1 Area under the curve: impact of resistive load

**FIGURE 4:**
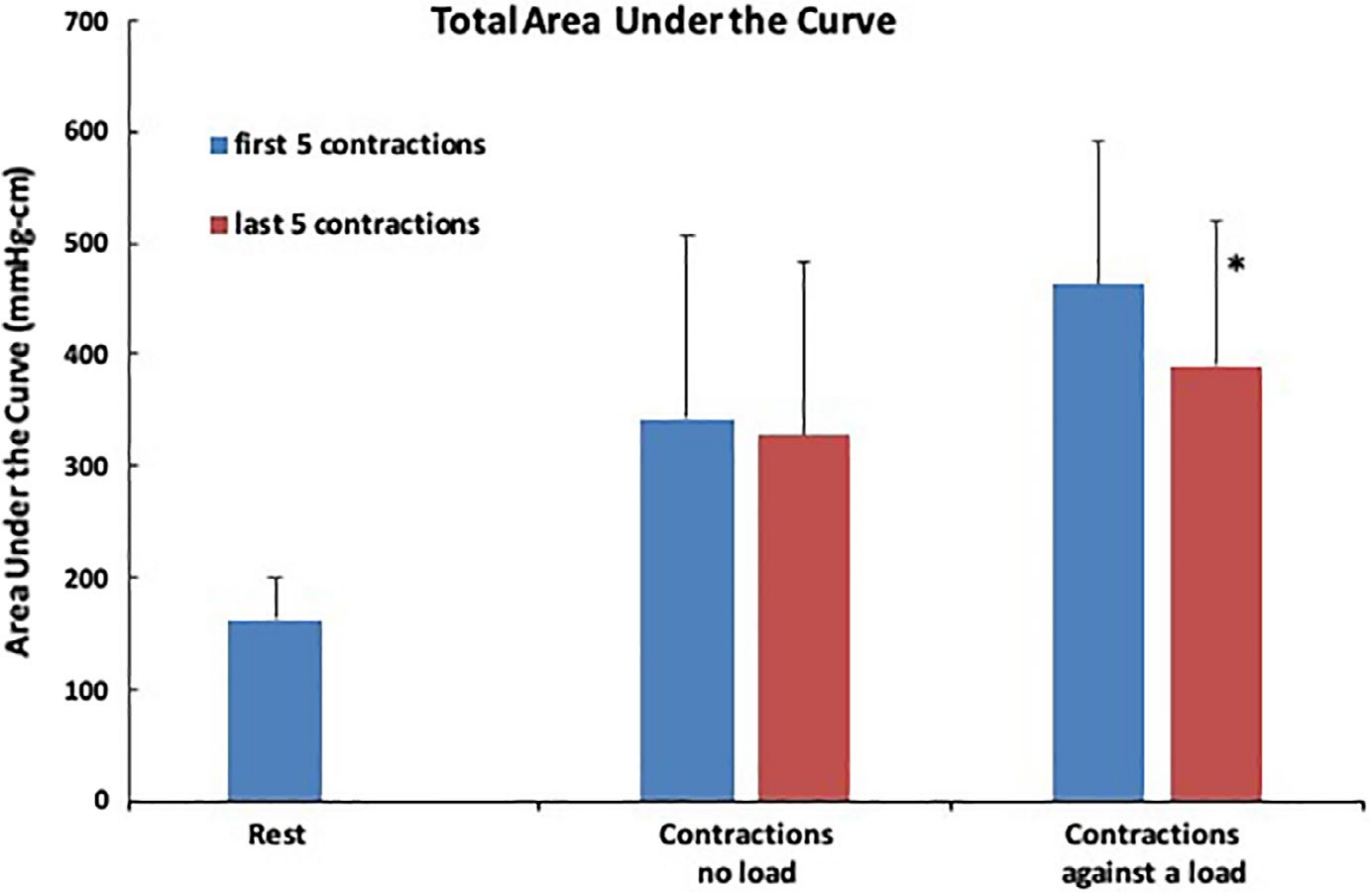
Total area under the curve (mmHg‐cm) during anorectal manometry for the resting state, contractions without a load, and contractions against a load for both the first five and last five contractions (±SD). * indicates statistical significance at *p* < 0.05 when comparing the first five to the last five contractions against a load using a paired *t*‐test. This indicates fatigue. There is no statistical difference when comparing the first five to last five contractions without a load. *n* = 12 subjects

During anal contractions, the anal pressure increased simultaneously at all sensor levels. The AUC was thus used as a metric of pressure throughout the anal canal at the time point when the strongest contraction occurred (mmHg‐cm). The AUC increased significantly during contractions regardless of the exercise condition when compared to the resting state (*p* < 0.05). When comparing the first 5 contractions (462 ± 129 mmHg‐cm) to the last 5 contractions (390 ± 131 mmHg‐cm) during 40 repetitive short squeeze contractions against a resistive load, the total AUC was significantly decreased indicating muscle fatigue (*p* = 0.02, Figure 4). However, there was no significant change in the total AUC during 40 repetitive short squeeze contractions without applying a resistive load (342 ± 164 mmHg‐cm, without vs. 329 ± 155 mmHg‐cm, with, *p* = 0.4).

#### 3.1.2 Caudal and rostral halves

**FIGURE 5:**
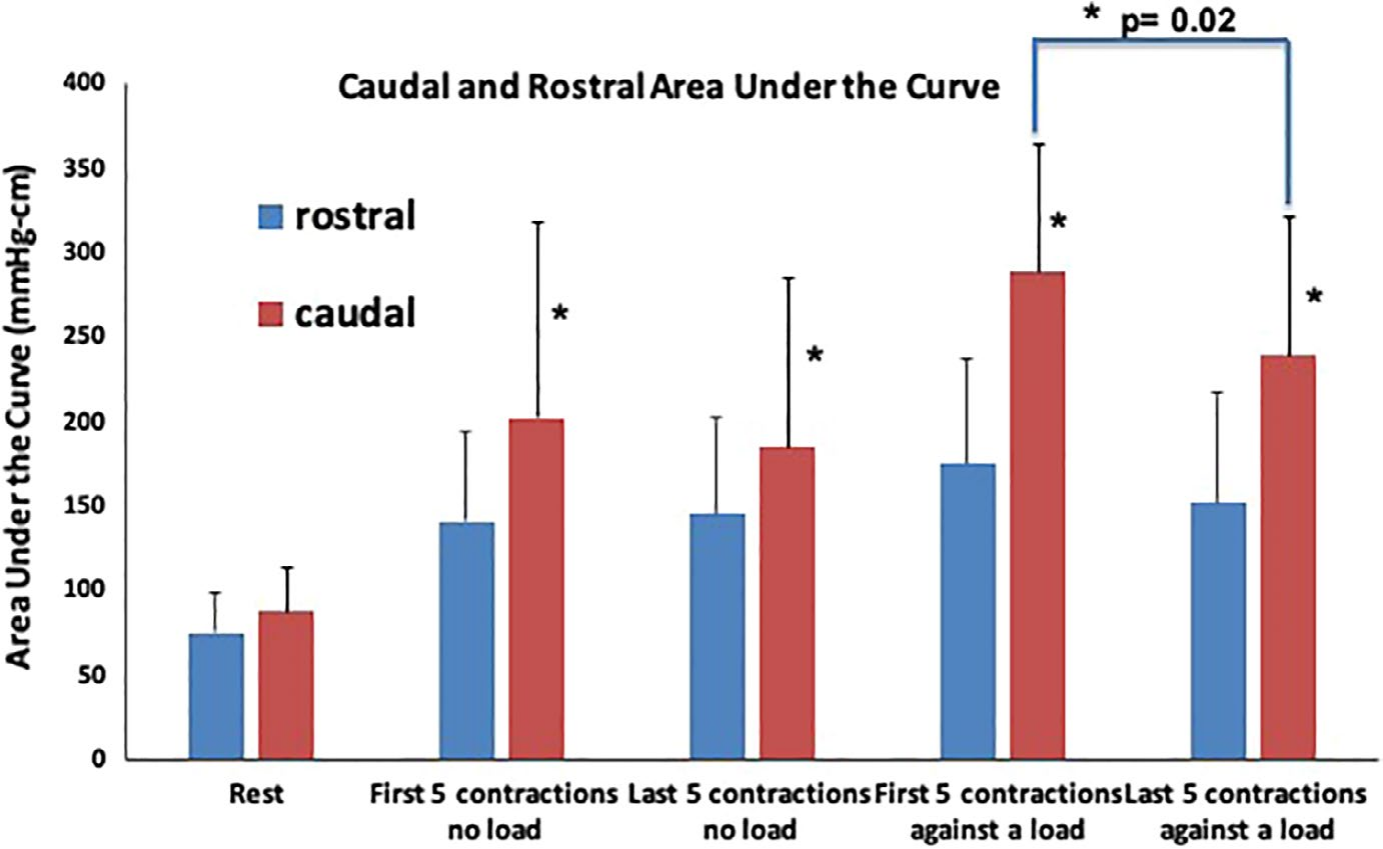
Caudal and rostral halves of area under the curve (AUC) during anorectal manometry for the resting state, contractions without a load, and contractions against a load for both the first five and last five contractions (±SD). * indicates statistical significance at *p* < 0.05 when comparing the rostral and caudal AUC halves using a paired *t*‐test. There is a significant difference between the rostral and caudal halves during contractions in all exercise settings. There is also a significant difference when comparing the caudal AUC for the first five to the last five contractions. *n* = 12 subjects

The caudal and rostral halves of the AUC were compared under different exercise conditions. In the resting state, there was no statistical difference between the caudal and rostral halves of AUC (87 ± 25 vs. 75 ± 23 mmHg‐cm, *p* = 0.2). However, during anal contraction, the caudal half AUC was consistently greater than the rostral half regardless of applying a resistive load (*p* < 0.05) (Figure 5). When comparing the first five to the last five contractions during repetitive short squeeze contractions against a resistive load, the caudal half of the AUC decreased significantly (288 ± 75 vs. 239 ± 82 mmHg‐cm, *p* = 0.02, Figure 5). In contrast, the difference was not significant when performing anal short squeeze contractions without applying anal load (202 ± 115 mmHg‐cm vs. 184 ± 101 mmHg‐cm, *p* = 0.2, Figure 5). The rostral half did not show any significant change in the AUC during anal short squeeze contractions in either condition.

### 3.2 Group 2—vaginal manometry, with and without resistive load

**FIGURE 6:**
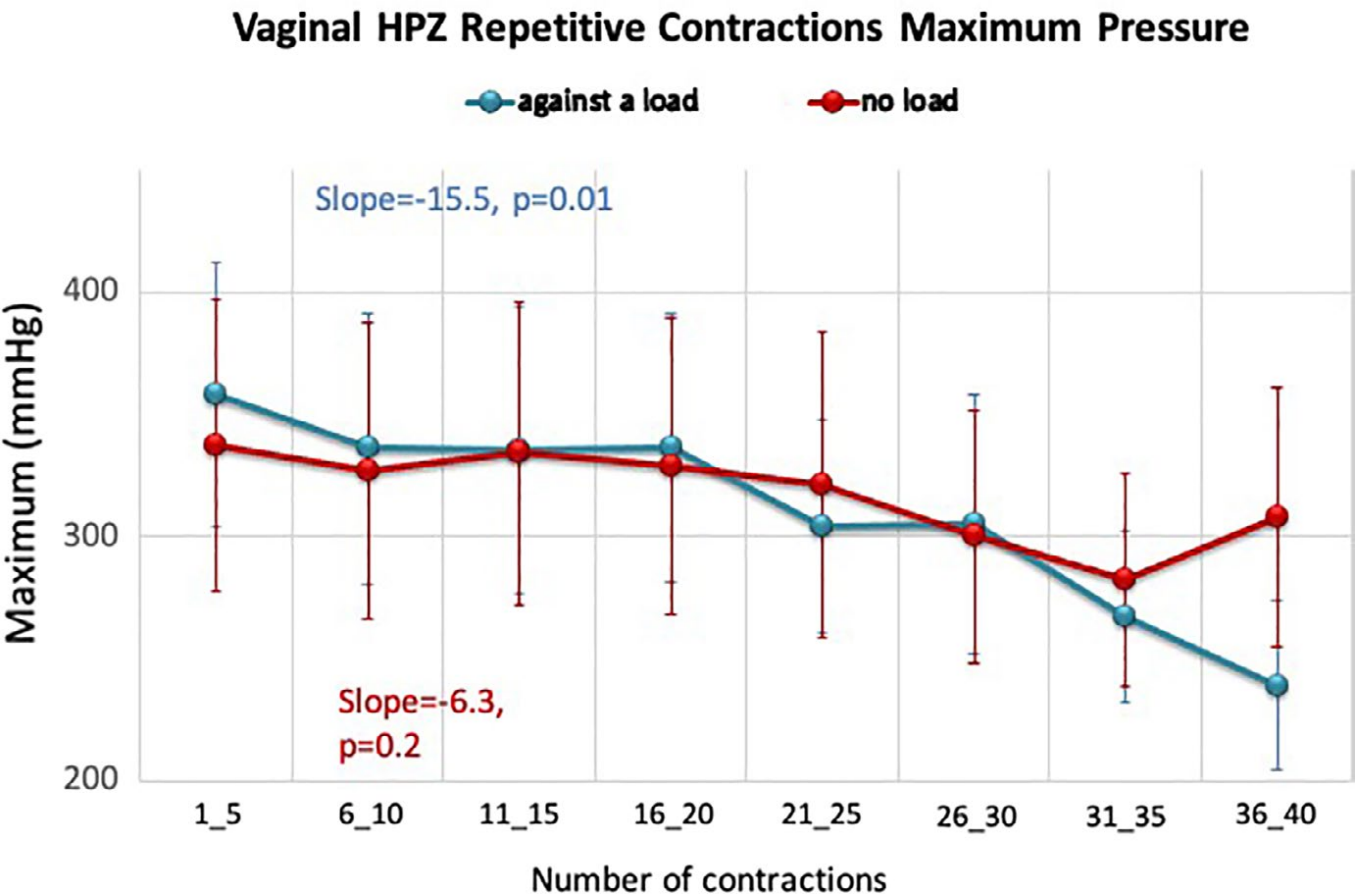
Change in maximum squeeze pressure (Max ± SD) in vaginal HPZ over 40 repetitive anal squeeze contractions, no load versus with load. The *y*‐axis is the maximum squeeze pressure (mmHg) and the *x*‐axis is the order of contractions, which are grouped into eight epochs. Repetitive short squeeze contractions against a load showed significant decrease in maximum squeeze pressure in vaginal HPZ. Linear regression analysis showed a significant negative correlation between maximum squeeze pressure and successive squeeze exercise against a load. There is no significant change in maximum squeeze pressure during exercise without load. *n* = 10 subjects

There was no significant change in the vaginal HPZ maximum (337 ± 189 vs. 308 ± 167 mmHg) or mean (273 ± 151 vs. 246 ± 111 mmHg) squeeze pressure during exercises without resistive load when comparing epoch 1 versus epoch 8. In contrast, anal contractions with the resistive load showed a significant decrease in vaginal HPZ maximum pressure (358 ± 171 vs. 239 ± 109 mmHg, *p* < 0.05) and mean pressure (318 ± 158 vs. 212 ± 96 mmHg, *p* < 0.05). Muscle fatigue was demonstrated by the significant negative correlation between successive contractions and weakening vaginal HPZ maximal squeeze pressure on linear regression analysis (Figure 6; slope with resistive load −15.5, *p* = 0.01 vs. slope without resistive load −6.3, *p* = 0.2). Similar findings were observed for the mean squeeze pressure (slope with cRED −14.7, *p* = 0.004 vs. slope without cRED −5.5, *p* = 0.2). Significant decrease of both maximum and mean squeeze pressures started at epoch 7, suggesting that fatigue occurred after subjects completed nearly 30 repetitive anal contractions.

## 4 DISCUSSION

In this study, we used HRAM and vaginal manometry to assess fatigability of the anal continence complex. Fatigability was demonstrated on both modalities only when using resistive loads. We used the AUC as the major metric of contractility as opposed to the previously used contractile integral (CI) on anorectal manometry as it allowed us to evaluate the longitudinal symmetry of the anal canal HPZ at the maximal squeeze point and compare the cumulative pressure changes under different exercise conditions. The total AUC and caudal half (anatomically correlating with EAS) decreased significantly during fatigue induced by repetitive contractions against a load. This result is consistent with our previous finding of anal sphincter fatigue induced by repetitive squeezes against a resistive load using the CI as a metric. While the rostral portion (anatomically the PRM) did not show fatigability on anorectal manometry, fatigability was seen on vaginal manometry. We feel that this demonstrates that vaginal manometry may offer more clarity in evaluation of the PRM’s contribution to continence than traditional anorectal manometry. In addition, we found that the PRM requires more repetitive contractions against a load to fatigue than the EAS.

The results of this study support our understanding of the physiologic changes in the anal sphincter muscles during repetitive short squeeze contractions. Our results corroborate previous reports regarding asymmetry in the anal canal HPZ by showing a greater pressure augmentation in the caudal compared to the rostral half of the HPZ during an anal contraction (Raizada et al., 2011).

Since the EAS and PRM are striated muscles, both are amenable to strengthening exercise. Our anorectal manometry results showed that the caudal half of the anal canal where the EAS predominates is more fatigable. This confirms that repetitive anal squeeze exercise against a resistive load is an effective strengthening exercise regimen for the EAS. However, the rostral part of the anal canal involves the PRM and appears more complex. We did not observe fatigue in the rostral half of the anal canal after repetitive short squeeze contractions when measuring the AUC with anorectal manometry. A possible explanation for this lack of observed fatigue is that anatomically the EAS contributes a smaller rostral pressure augmentation than to the caudal part. Though there are studies suggesting voluntary contractions of the PRM and EAS increase pressure in the proximal and distal halves of the anal canal (Liu et al., 2006), respectively, the current anorectal manometry technique may not be sensitive enough to identify the definite activity from the PRM.

We aimed to confirm the ability to fatigue the PRM. The vaginal HPZ has been described previously as reflecting the contraction of the PRM (Raizada et al., 2010), which provides a unique way to evaluate the function of this muscle. We thus evaluated fatigue of the PRM by assessing the change in the vaginal HPZ during various anal squeeze exercises. We found the PRM could be fatigued more effectively with the application of the cRED during exercise. We observed fatigue occurred after about 30 contractions, which is different from our prior study that showed fatigue started after 20 contractions when anal canal pressure changes were used to assess fatigue (Mei et al., 2021). These results suggest that both the EAS and PRM are fatigable, however compared to the EAS, the PRM requires more repetitions of short squeeze contractions against resistance to achieve fatigue. We speculate this difference could be due to variations in anatomical orientation, muscle mass, and muscle fiber composition.

PFM exercises are more efficient in inducing fatigue when there is incorporation of resistance provided by a load like the cRED. The pressure augmentation during squeeze and the subsequent induced fatigue after repetitive contractions are unevenly distributed longitudinally along the anal canal. The findings also show that the PRM fatigues at a slower rate than the EAS, which holds clinical ramifications on the rehabilitation of the continence complex.

Our study has limitations in its applicability to women with fecal incontinence as the primary aim of this study was to better understand normal exercise physiology of the anal sphincter complex in rehabilitative training. We understand multiple factors including age, gender, history of trauma, and other medical comorbidities can affect the physiology of fecal continence. Future studies will expand to different populations including postpartum and elderly women. Another limitation is that our two assessment techniques were not studied in the same women which limits our ability to compare the outcomes. However, we hope that this limitation was mediated by utilizing only healthy, nulliparous women, and using within‐subject comparisons. Another limitation of this study is that we only utilized manometry in our study which cannot provide definite information on the anorectal anatomy. Future studies should also include assessments of anorectal anatomy including ultrasound, defecography, and dynamic pelvic MRI to obtain structural details during anal resistance exercise to compare the findings with the manometry results. Strengths of this study include the use of multiple methods in assessing fatigue of the continence complex, randomization of the exercise sequence, and the same interpreters throughout the study.

In summary, the pressure profile of the manometric anorectal HPZ is asymmetric. Axial asymmetry has been previously described during a contraction. However, this is the first study to show asymmetry in a fatigued contraction due to a resistive load. Different continence muscles, including the EAS and PRM, exhibit fatigue by contractions against a load. This was evidenced by reduction of the caudal half of the AUC and squeeze pressures of the vaginal HPZ. These findings pave the way for future studies in understanding exercise physiology, provide specific targets for interventions that could strengthen the fecal continence complex more effectively, and may guide the development of appropriate exercise regimens to target distinct muscles involved in fecal continence.

## CONFLICT OF INTEREST

The authors report no financial or personal conflict of interest.

## AUTHOR CONTRIBUTIONS

K. P. had a major role in the study concept and design, acquisition and interpretation of the data, statistical analysis, and the preparation of the manuscript. L. M. had a major role in the study concept and design, acquisition and interpretation of the data, statistical analysis, and the preparation of the manuscript. E.Y. had a major role in interpretation of the data, statistical analysis, and the preparation of the manuscript. M. K had a major role in interpretation of the data, statistical analysis, and the preparation of the manuscript. N. L. had a major role in subject recruitment and data acquisition. F. E. had a major role in the study concept and design, interpretation of the data, and the preparation of the manuscript. P. S. had a major role in the study concept and design, interpretation of the data, and the preparation of the manuscript. E. D. had a major role in the study concept and design, interpretation of data, and the preparation of the manuscript. R. S. had a major role in the study concept and design, interpretation of data, the preparation of the manuscript, funding and supervision of the project, and development of the manuscript.
